# Oxygen reduction reaction activity of an iron phthalocyanine/graphene oxide nanocomposite†

**DOI:** 10.1039/d1ra01001h

**Published:** 2021-04-29

**Authors:** Kenichiro Irisa, Kazuto Hatakeyama, Soichiro Yoshimoto, Michio Koinuma, Shintaro Ida

**Affiliations:** Graduate School of Science and Technology, Kumamoto University 2-39-1 Kurokami, Chuo-ku Kumamoto 860-8555 Japan; Institute of Industrial Nanomaterials (IINa), Kumamoto University 2-39-1 Kurokami, Chuo-ku Kumamoto 860-8555 Japan ida-s@kumamoto-u.ac.jp

## Abstract

Electrocatalysts with metal–nitrogen–carbon (M–N–C) sites have recently attracted much attention as potential catalysts for the oxygen reduction reaction (ORR), and a hybrid of iron phthalocyanine (FePc) and reduced graphene oxide (rGO) is one of the promising candidates. Herein, a FePc/GO nanocomposite was synthesized by electrostatic deposition on the electrode. The electrochemically reduced FePc/GO nanocomposite (ER(FePc/GO)) contained Fe^2+^ centers in well reduced graphene sites without agglomeration. The ER(FePc/GO) exhibited high ORR activity with an ORR onset (*E*_onset_) and half-wave potential (*E*_1/2_) of 0.97 and 0.86 V, respectively. Furthermore, the ORR activity successfully improved by adding an electrolyte such as KCl or KNO_3_. The small H_2_O_2_ yield of 2%, superior tolerance to methanol addition and high-durability indicate that the ER(FePc/GO) is a promising electrocatalyst. Theoretical studies, indicating that the presence of Cl^−^ and NO_3_^−^ ions lowered the conversion energy barrier, strongly supported the experimental results.

## Abstract 2

We demonstrate from both experimental and theoretical viewpoints that an iron phthalocyanine/graphene oxide nanocomposite directly synthesized on electrode exhibits high ORR activity.

## Graphical abstract

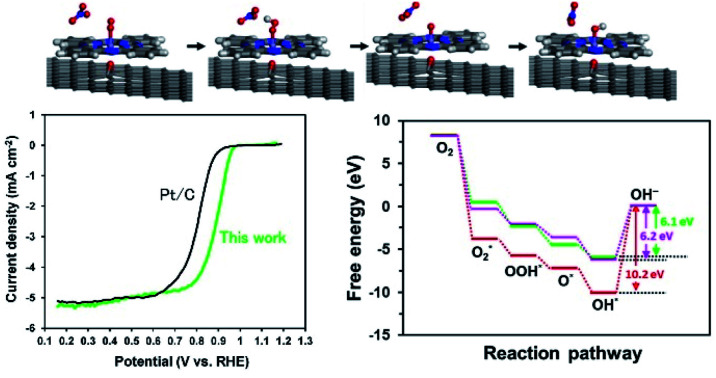

## Introduction

Graphene oxide (GO) is a function-tunable material whose electronic conductivity, ionic conductivity, bandgap, and hydrophilicity can be controlled through modification of its surface oxygen functional groups.^1–7^ Furthermore, the surface oxygen functional groups on GO enable the preparation of functional hybrid materials that interact face-to-face with various organic compounds.^8–12^ Such hybrid materials are desirable as oxygen reduction reaction (ORR) electrocatalysts that operate at the three-phase interface of hydrogen fuel cells and air batteries and require high current collection, good ionic conductivity, and a high specific surface area.^13–15^ In general, expensive Pt-based electrocatalysts have been used to promote the ORRs that occur at the cathode in fuel cells and air batteries. However, Pt-based electrocatalysts suffer from the scarcity and poor CO tolerance of Pt.^16–19^

Nanocomposites of reduced GO (rGO) and FePc have been reported to exhibit ORR activity superior to that of Pt/C.^20–24^ Metal–nitrogen–carbon (M–N–C) sites have attracted much attention as potential highly active single-atom catalysts, and an FePc/rGO nanocomposite, in which the Fe–N–C sites on FePc are nanoscale-adjacent to the graphene sites in rGO with high electron mobility clearly exhibits high ORR activity.^21,25,26^ However, ideal hybrids between monolayer GO nanosheets and FePc have not yet been achieved because previously reported FePc/GO nanocomposites were prepared using a poorly-dispersed FePc in ethanol (EtOH) or *N*,*N*-dimethylformamide (DMF) containing a large amount of aggregated FePc.^22,23^ An ideal monolayer FePc/GO hybrid is expected to exhibit greater catalytic activity than previously reported FePc/rGO composites.

In the present study, we report a composite that exhibits better ORR activity than any previously reported FePc/rGO nanocomposite. The new composite was prepared by electrostatic interaction between GO nanosheet and FePc on the electrode. Furthermore, we demonstrate from both experimental and theoretical viewpoints that the addition of electron-withdrawing ions to the electrolyte solution positively affects the ORR activity of the nanocomposite.

## Experimental

### Materials

All chemicals were obtained from commercial suppliers and were used without further purification. Iron phthalocyanine (FePc, 90%, Sigma-Aldrich), chloroform (99.0%, Wako), graphite powder (98.0%, Wako), and DMF (99.0%, Wako) were used.

### Synthesis of GO

GO was prepared using a modified Hummers' method.^27,28^ Graphite powder (2 g), NaNO_3_ (2 g) and H_2_SO_4_ (92 mL) was mixed at 0 °C and KMnO_4_ (10 g) was added to the mixture slowly. The resulting mixture was diluted with deionized water (DI), then 30% H_2_O_2_ (5 mL) was added, centrifuged, washed with 5% HCl solution and DI water, and dried in an oven at 50 °C for 2 days. The resultant graphite oxide was exfoliated in DI water by ultrasonication (KUBOTA 3700) for 2 h; the sonicated solution was subsequently centrifuged at 8000 rpm to separate the GO from graphite oxide. The obtained GO/water dispersion was dried at 50 °C to obtain GO powder.

### Preparation of electrocatalyst

A GO/DMF dispersion (0.5 g L^−1^) was prepared by sonicating the prepared GO powder in DMF for 2 h. An FePc/CHCl_3_ dispersion (0.5 g L^−1^) was prepared by sonicating FePc in CHCl_3_ for 1 h. FePc/GO was prepared by mixing 20 μL of the GO/DMF dispersion (0.5 g L^−1^) and 20 μL of the FePc/CHCl_3_ dispersion (0.5 g L^−1^) on a glassy carbon (GC) electrode. Furthermore, the FePc/GO was reduced electrochemically to ER(FePc/GO) by applying a potential of −1.3 V (*vs.* Ag/AgCl, pH 13) for 10 min to the GC electrode coated with FePc/GO in N_2_-saturated 0.1 M KOH.

### Materials characterization

The height and width of the resultant GO nanosheets were investigated by atomic force microscopy (AFM; Nanocute, Hitachi High-Tech Sci. Co.). X-ray diffraction (XRD) patterns were collected on a Rigaku SmartLab 3 kW X-ray diffractometer equipped with a Cu Kα (*λ* = 0.154 nm) radiation source. A field-emission Scanning electron microscopy (FE-SEM) images were recorded on a SU-8000 (Hitachi High-Technologies Corporation) microscope. Elemental analyses and chemical-state profiles were conducted using an X-ray photoelectron spectroscopy (XPS) apparatus (ThetaProbe, Thermo Fisher Scientific). The C 1s XPS spectra were fitted based on of the contributions of eight groups (–COOH, C=O, C–O–C, C–OH, sp^3^ C–C, C–H defects, C–N, and sp^2^ C=C bonds). Fe 2p_3/2_ and Fe 2p_1/2_ XPS spectra were fitted based on the contributions from four groups (Fe^2+^ + N, Fe^3+^ + N, Fe oxide, and Fe satellite).

### Electrochemical measurements

Electrochemical measurements were conducted at room temperature on an Autolab PGSTAT128N (Metrohm, Netherlands) with a rotating-disk electrode (RDE) and a rotating ring-disk electrode (RRDE) system (PINE, Inc.). A GC RDE (diameter: 5 mm; geometric area: 0.196 cm^2^) coated with an electrocatalyst, an Ag/AgCl electrode in saturated KCl, and a Pt plate electrode (0.8 cm^2^) were used as the working, reference, and counter electrodes, respectively. The potential *vs.* Ag/AgCl was converted to the reversible hydrogen electrode (RHE) scale by the following equation:

1 *E*_(*vs.* RHE)_ = *E*_(*vs.* Ag/AgCl)_ + 0.197 + 0.059 × pH

The ER(FePc/GO) electrocatalyst was prepared by applying a potential to the FePc/GO (GO 10 μg, FePc 10 μg) on a GC electrode in N_2_-saturated 0.1 M KOH, as already mentioned (resulting in an Fe loading of 0.090 mol_Fe_ cm^−2^). For the comparison, an ink containing a commercial Pt/C catalyst was prepared by sonicating 5 mg of 20 wt% Pt/C (EC-20, Pt on Vulcan XC-72, ElectroChem, Inc), 40 μL of 5 wt% Nafion suspension (Wako), and 1.66 mL of ethanol (99.5%, Wako) for 1 h and then dropping 5 μL of the resultant suspension onto a GC electrode (resulting in a Pt loading of 0.077 mol_Pt_ cm^−2^).

The cyclic voltammetry (CV) and RRDE measurements were conducted at a scanning rate of 10 mV s^−1^ in an O_2_-saturated 0.1 M KOH solution, a 0.1 M KOH solution containing an electrolyte such as KCl or KNO_3_. The tolerance test toward methanol was conducted at 0.8 V (*vs.* RHE) in O_2_-saturated 0.1 M KOH at 500 rpm using a chronoamperometric method. A stability test of the as-prepared catalysts for ORR was conducted at 0.5 V (*vs.* RHE) in O_2_-saturated 0.1 M KOH at 1600 rpm using a chronoamperometric method.

The hydrogen peroxide yields (H_2_O_2_%) and the electron transfer number (*n*) were calculated from the RRDE data using the equations2
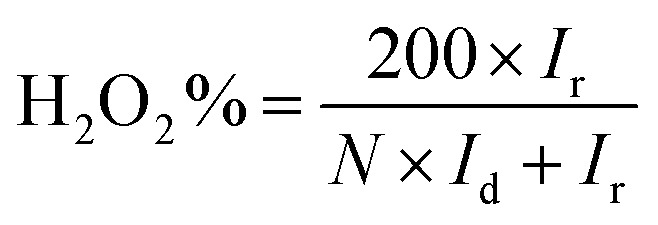
3
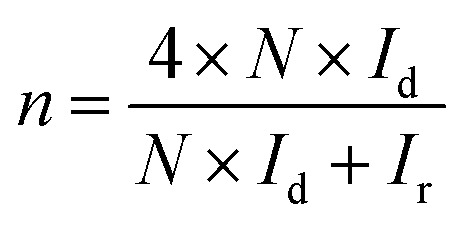
where *I*_d_ is the disk current, *I*_r_ is the ring current, and *N* = 0.25 is the collection efficiency of the Pt-ring electrode.

### Theoretical calculations

Density functional theory (DFT) calculations were performed using the Cambridge Sequential Total Energy Package (CASTEP) program in the Materials Studio software package.^29^ The generalized gradient approximation of Perdew–Burke–Ernzerhof was used for the exchange–correlation potential; a plane-wave kinetic energy cutoff of 400 eV was used here.^30^ van der Waals (vdW) forces were corrected using the D2 method of Grimme.^31^ The convergence criterion was set to 0.05 eV Å^−1^ for the force and 10^−5^ eV per atom for the energy. We applied an exchange energy (*J*) of 1 eV for Fe 3d orbitals.^32^

In the present study, the structure of an O atom on a graphene framework and an FePc molecule on top of it was regarded as ER(FePc/GO).

## Results and discussion

### Structure of GO

**Fig. 1:**
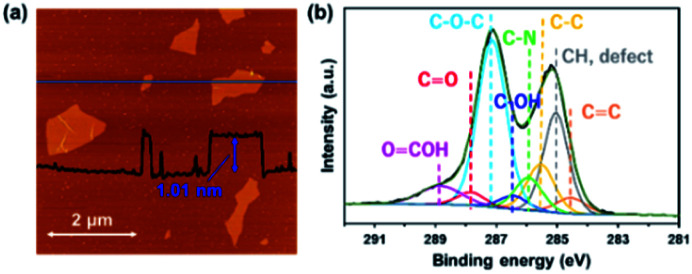
(a) AFM image and (b) the C 1s XPS spectrum of a GO sheet exfoliated in DMF.

Fig. 1a shows an AFM image of GO sheets exfoliated in DMF. The thickness of the GO sheet was ∼1.0 nm, indicating that GO was completely exfoliated to single layers in the DMF. Fig. 1b shows the C 1*s* XPS spectrum of a GO sheet. The spectrum of the GO sheet shows two main peaks at 285.1 and 287.1 eV; the former is derived from the graphene domain, and the latter arises from O-containing functional groups. The degree of oxidation (O/(C + O)) was 0.36, which indicates that GO was sufficiently oxidized. Peaks in the C 1s spectra were assigned to various O-containing functional groups (*i.e.*, C=O, C–O–C, and COOH) and a C=C group. The GO sheets were negatively charged in the DMF solution because of their O functional groups, which enabled the formation of a nanohybrid between the GO sheets and the FePc *via* electrostatic interactions and π–π interactions between the FePc and the O atoms in the GO.^22^

### Structure of FePc/GO and ER(FePc/GO)

**Fig. 2:**
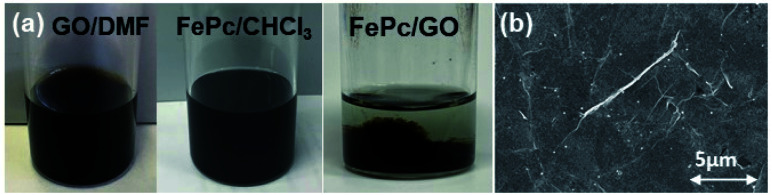
(a) Photographs of GO/DMF dispersions, an FePc/CHCl_3_ dispersion, and a mixture of these two dispersions. (b) SEM image of ER(FePc/GO).

Fig. 2a shows photographs of a GO-dispersed DMF solution (GO/DMF), an CHCl_3_ solution with dissolved FePc (FePc/CHCl_3_), and a mixture of these two dispersions. The single layer GO was well dispersed in DMF without aggregating, and the FePc/CHCl_3_ exhibited high dispersion stability, remaining dispersed even after 1 month. In a previous study, methanol and/or DMF were used to dissolve FePc.^22,23^ The state of dispersion of FePc in methanol and DMF was unstable, therefore, FePc dispersed EtOH and/or DMF was precipitated at the bottom after 1 day (see Fig. S1, ESI†). Because of strong interaction between the GO and FePc, the FePc/GO nanocomposite was immediately produced when the GO/DMF and FePc/CHCl_3_ solutions were mixed. The FePc/GO nanocomposite was directly prepared on the GC electrodes. The FePc/GO composite was electrochemically reduced at −0.8 V *vs.* Ag/AgCl in a KOH aqueous solution. The electrochemically reduced FePc/GO composite was denoted as ER(FePc/GO). Fig. 2b shows an FE-SEM image of the ER(FePc/GO) on the electrode. Only GO-derived wrinkle structure was observed in the SEM image, indicating that the FePc molecules were adsorbed uniformly on the rGO surface.

**Fig. 3:**
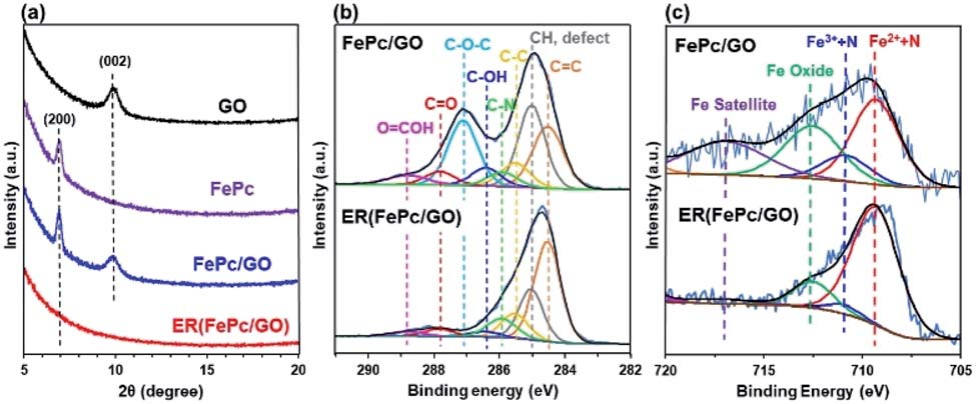
(a) XRD patterns of GO, FePc, GC-plate FePc/GO, and ER(FePc/GO); (b) C 1s and (c) Fe 2p_3/2_ XPS spectra of FePc/GO and ER(FePc/GO) on a GC plate.

The XRD patterns of the GO, FePc, FePc/GO, and ER(FePc/GO) on a GC plate are shown in Fig. 3a. The strongest XRD peaks of GO and FePc appeared at ∼9.9° and ∼7.0°, corresponding to the (002) planes of GO and the (200) planes of FePc, respectively. The XRD pattern of FePc/GO shows peaks attributable to both GO and FePc, indicating that restacked GO and recrystallized FePc were present in the FePc/GO. After electrochemical reduction, the peaks attributed to GO and FePc disappeared. Their disappearance is attributed to the desorption of residual restacked GO and recrystallized FePc; a composite with a dense structure at the nanolevel was produced.

XPS measurements were performed to investigate the chemical states of the GO and Fe in the FePc/GO and ER(FePc/GO) nanocomposites. Fig. 3b shows the C 1*s* spectra of FePc/GO and ER(FePc/GO). The intensities of the peaks of the epoxy groups (287.1 eV) in FePc/GO greatly decreased after the electrochemical reduction process, indicating that GO was successfully reduced. The reduction of GO enables the electrocatalyst to attain the high electrical conductivity required for high catalytic activity. Fig. 3c shows the Fe 2p_3/2_ spectra of FePc/GO and ER(FePc/GO). A comparison of these spectra reveals that the Fe^3+^ and Fe^2+^ peaks of ER(FePc/GO) are less intense and more intense, respectively, than those of FePc/GO. This result indicates that the Fe^3+^ was reduced to Fe^2+^ under the applied potential. Fe^2+^ generally exhibits greater activity than Fe^3+^ toward the ORR.^22^ We therefore expected the ER(FePc/GO), which exhibits high electrical conductivity and contains Fe^2+^, to demonstrate high ORR activity.

### Catalytic activities of FePc/GO and ER(FePc/GO)

**Fig. 4:**
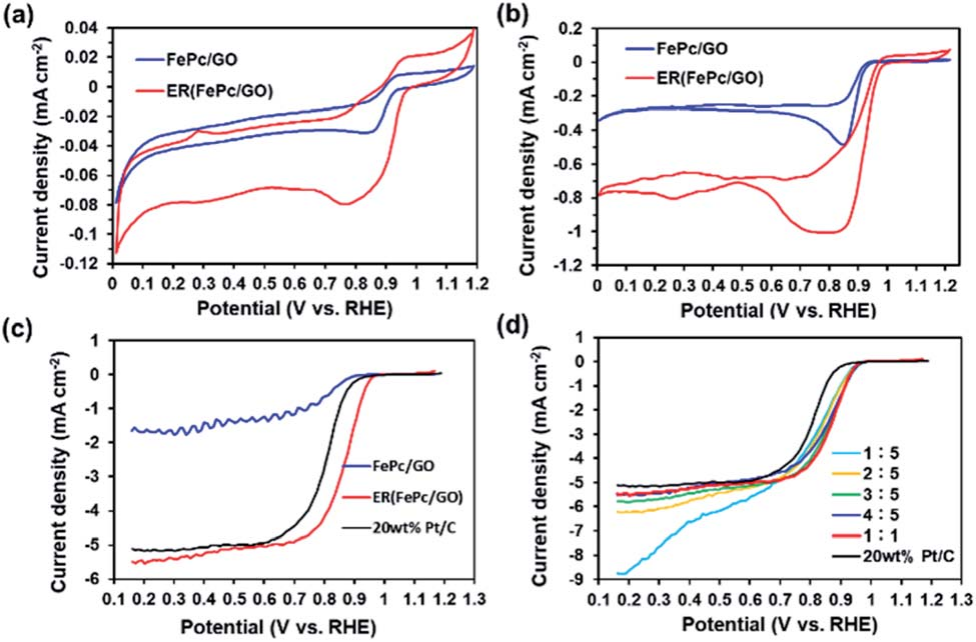
Cyclic voltammograms of FePc/GO and ER(FePc/GO) in (a) N_2_- and (b) O_2_-saturated 0.1 M KOH solutions. (c) ORR polarization curves of FePc/GO, ER(FePc/GO), and 20 wt% PtC in O_2_-saturated 0.1 M KOH, as a recorded at an electrode rotation rate of 1600 rpm and a scan rate of 10 mV s^−1^. (d) RDE measurements of ER(FePc/GO) mixed with FePc and GO in various ratios, as recorded using O_2_-saturated 0.1 M KOH, an electrode rotation rate of 1600 rpm, and a scan rate of 10 mV s^−1^.

To investigate the ORR activity of FePc/GO and ER(FePc/GO), CV measurements were performed in 0.1 M KOH solution with N_2_ or O_2_ aeration. Fig. 4a and b shows the current–voltage (*I*–*V*) curves for the FePc/GO and ER(FePc/GO) nanocomposites. The cyclic voltammograms of the FePc/GO and ER(FePc/GO) nanocomposites in the N_2_-saturated 0.1 M KOH solution (Fig. 4a) show only redox peaks of Fe^3+^ and Fe^2+^. However, the cyclic voltammograms of the same materials in the O_2_-saturated 0.1 M KOH solution (Fig. 4b) show negative currents, which we attributed to the ORR. The onset potentials (*E*_onset_) of the FePc/GO and ER(FePc/GO) were 0.92 V and 0.98 V (*vs.* RHE), respectively. The ER(FePc/GO) showed a higher *E*_onset_ than the FePc/GO because of its higher electrical conductivity and greater Fe^2+^/Fe^3+^ ratio. The high ORR activity of the ER(FePc/GO) composite was confirmed *via* RDE experiments. The ORR polarization curves in Fig. 4c reveal that the ORR onset and half-wave potential of ER(FePc/GO) are 0.97 and 0.86 V, which are greater those of the commercial 20 wt% Pt/C (*E*_onset_ = 0.91 V, *E*_1/2_ = 0.81 V). The ER(FePc/GO) nanocomposite containing FePc and GO in a 1 : 1 ratio exhibited the highest ORR activity, as shown in Fig. 4d.

### Effect of adding electrolyte on the ORR activity

**Fig. 5:**
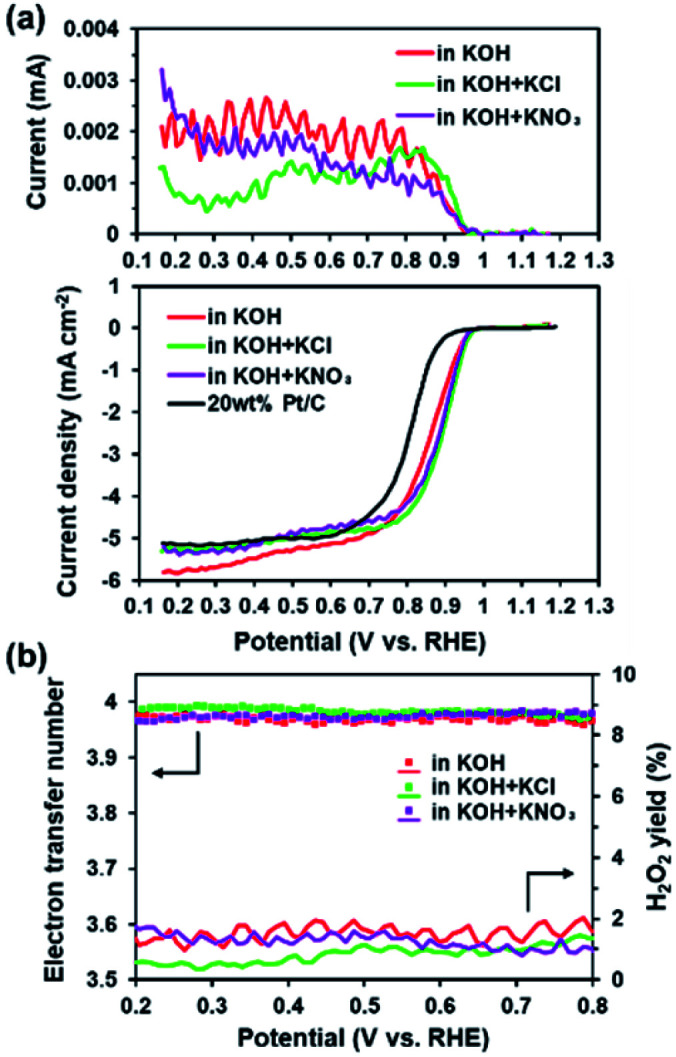
(a) RRDE measurements of the ER(FePc/GO) nanocomposite in O_2_-saturated 0.1 M KOH and 0.1 M KOH containing various electrolytes; the measurements were conducted with a rotation rate of 1600 rpm and at a scan rate 10 mV s^−1^. (b) Electron transfer numbers and percentage H_2_O_2_ yield of ER(FePc/GO) in O_2_-saturated 0.1 M KOH and 0.1 M KOH containing various electrolytes.

The presence of electron-withdrawing ions near FePc leads to high ORR activity because of a decrease in the electron density of Fe atoms.^33,34^ Therefore, ORR measurements of the ER(FePc/GO) composite were performed after an electrolyte such as KCl or KNO_3_ was added to the 0.1 M KOH solution. The ORR polarization curves in Fig. 5a show that the ORR onset and half-wave potential positively shifted upon addition of KCl or KNO_3_. These results clearly indicate that the ORR activity was improved by the addition of KCl or KNO_3_ to the electrolyte. The average electron transfer number of ER(FePc/GO) in 0.1 M KOH solutions with KCl and KNO_3_ was 3.97 and 3.98, respectively. This result indicates that the reduction of O_2_ to OH^−^ over ER(FePc/GO) occurred *via* a four-electron transfer process. The H_2_O_2_ yield was smaller than 2% in the potential range from 0.2 to 0.8 V at all of the samples, as shown in Fig. 5b, which indicates low peroxide formation during the ORR.

**Fig. 6:**
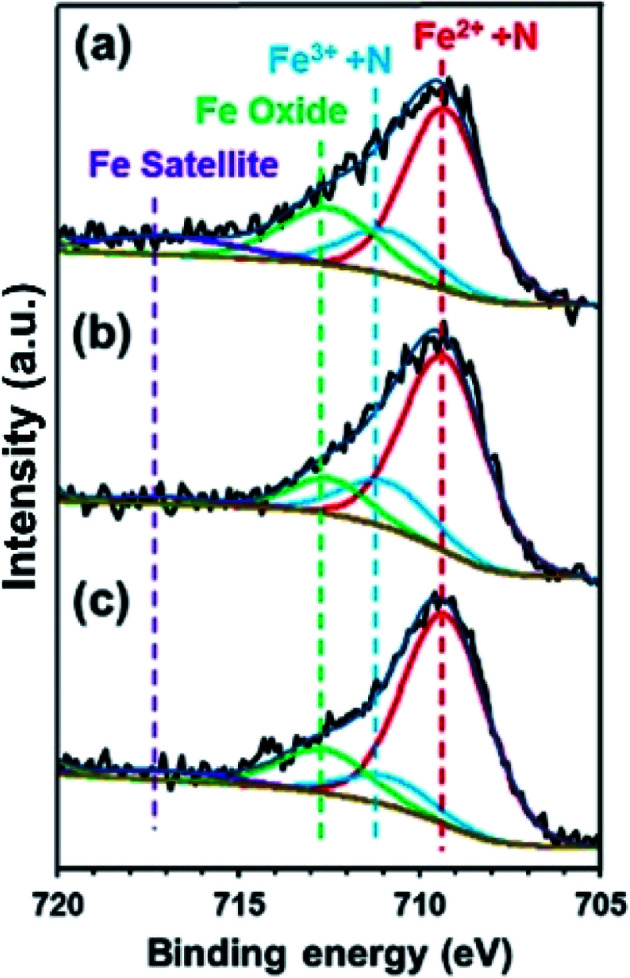
Fe 2p_3/2_ spectra of ER(FePc/GO) subjected to an applied potential (0.57 V *vs.* RHE, 10 min) in (a) 0.1 M KOH solution, (b) 0.1 M KOH solution containing 0.1 M KCl, and (c) 0.1 M KOH solution containing 0.1 M KNO_3_.

To confirm the effect of Cl^−^ and NO_3_^−^ anions on the electron density of the Fe in FePc, XPS measurements were performed on the ER(FePc/GO) nanocomposite after a potential was applied in each solution saturated with O_2_; the results are shown in Fig. 6. The Fe^2+^/Fe^3+^ ratio of ER(FePc/GO) subjected to an applied potential (0.57 V *vs.* RHE, 10 min) in 0.1 M KOH solution, 0.1 M KOH solution containing 0.1 M KCl, and 0.1 M KOH solution containing 0.1 M KNO_3_ is 3.7, 4.0, and 7.0, respectively. These results show that the presence of an electrolyte in the KOH solution inhibits the oxidation of Fe^2+^, enabling it to remain in the more highly active divalent state.

**Fig. 7:**
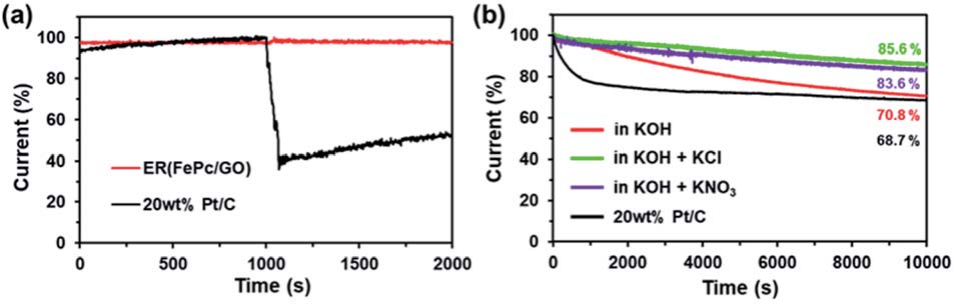
(a) Tests of the methanol tolerance of ER(FePc/GO) in 0.1 M KOH and in 0.1 M KOH with various electrolytes at 0.5 V (*vs.* RHE) and 500 rpm. (b) Current–time (*i*–*t*) curves of ER(FePc/GO) at 0.5 V (*vs.* RHE) in O_2_-saturated 0.1 M KOH and 0.1 M KOH with various electrolytes at 1600 rpm.

When 3.0 M methanol was added to the 0.1 M KOH electrolyte solution, ER(FePc/GO) showed a stable amperometric response without a substantial loss in current density (Fig. 7a), indicating a superior tolerance to methanol addition. In contrast, the commercial 20 wt% Pt/C showed a 60% decrease in current after methanol addition. This result suggests that ER(FePc/GO) could also be used as a methanol-tolerant cathode catalyst in direct methanol fuel cells.

In addition to activity, durability is a major factor affecting the suitability of electrocatalysts for use in fuel cells. As shown in Fig. 7b, the current of ER(FePc/GO) at 0.5 V (*vs.* RHE) decreased to 70.8% after 10 000 s in 0.1 M KOH at a rotation rate of 1600 rpm, which represents greater durability than that of the 20 wt% Pt/C. When an electrolyte was added to the KOH solution, the durability was strongly affected: the ER(FePc/GO) maintained better than 80% of its initial current. This result is attributed to the oxidation of Fe in FePc being suppressed by the electrolyte.

### Theoretical calculations

**Fig. 8:**
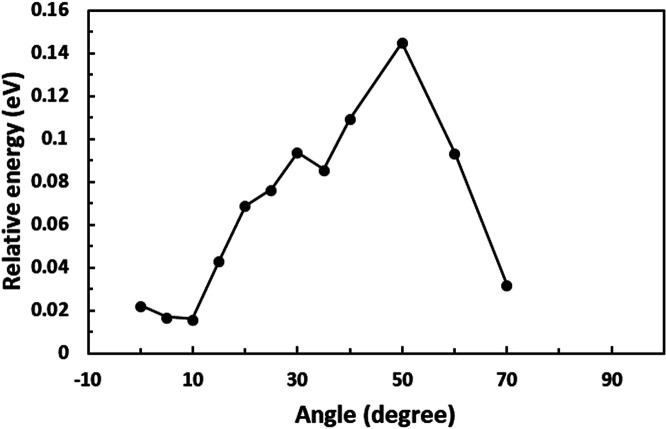
Optimization of the installation angle of FePc to graphene.

**Fig. 9:**
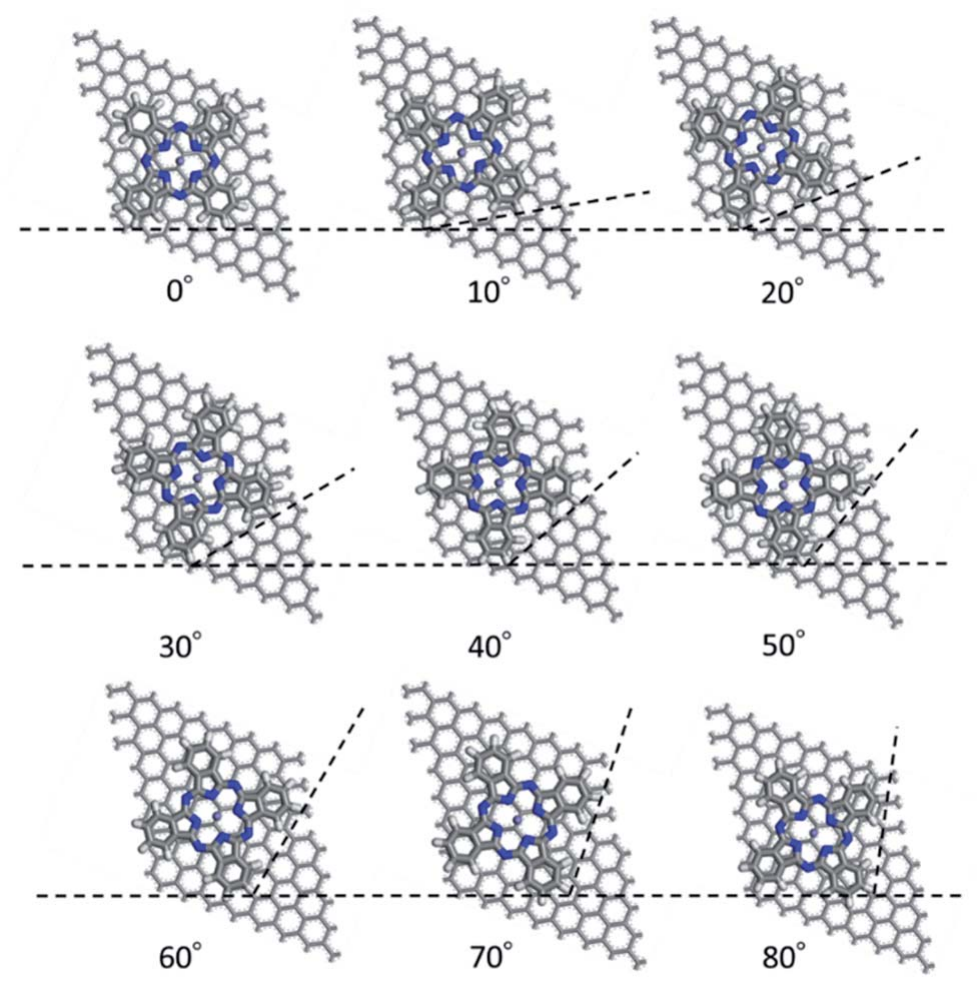
Structure of ER(FePc/GO) depending on the installation angle of FePc to the graphene framework.

First, we optimized the structure of ER(FePc/GO) using DFT calculations. A graphene framework with a C–O–C oxygen functional group placed on it was considered as reduced GO, and an FePc molecule was placed on the O atom. Structural optimization showed that the distance between Fe and O was 1.962 Å (see Fig. S4, ESI†). The angle of FePc to the graphene framework was also optimized as shown in Fig. 8 and 9. The total enthalpy was depended on the angle of FePc to the graphene framework, and the structure 10° was the most stable structure among the nine structure shown in Fig. 9, where the lamination deviations were observed between the four 6-membered carbon rings of the FePc and the 6-membered carbon rings of the GO surface. Next, the DFT calculations to analyze the ORR mechanism of ER(FePc/GO) under alkaline conditions with and without electron-withdrawing groups such as Cl^−^ were performed. The 4e^−^ pathway of the ORR in the alkaline medium is expressed as follows:^35^

Overall reaction: O_2_ + 2H_2_O + 4e^−^ → 4OH^−^

1 * + O_2_ + 2H_2_O + 4e^−^ → *O_2_ + 2H_2_O + 4e^−^

2 *O_2_ + 2H_2_O + 4e^−^ → *OOH + (OH)^−^ + H_2_O + 3e^−^

3 *OOH + (OH)^−^ + H_2_O + 3e^−^ → *O + 2(OH)^−^ + H_2_O + 2e^−^

4 *O + 2(OH)^−^ + H_2_O + 2e^−^ → *OH + 3(OH)^−^ + e^−^

5 *OH + 3(OH)^−^ + e^−^ → * + 4(OH)^−^

Here, * denotes the active site of FePc.

**Fig. 10:**
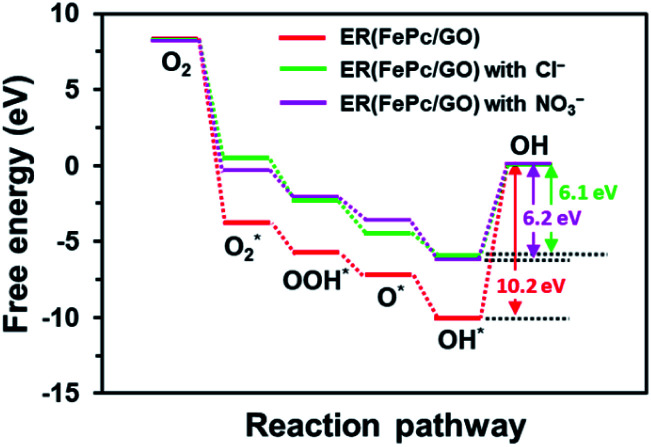
Free energy diagram of ORR for ER(FePc/GO).

**Fig. 11:**
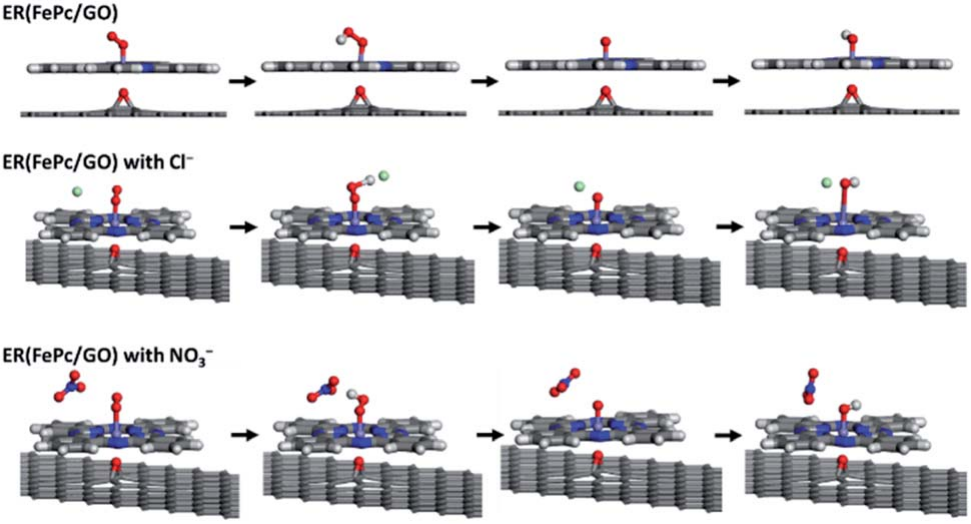
Optimized structure of ER(FePc/GO) along the ORR reaction pathway.

The free-energy diagrams of ER(FePc/GO) in an alkaline medium with and without Cl^−^ from the reaction (1) to (5) are shown in Fig. 10. Fig. 11 shows the structures corresponded to the each step in the energy diagrams in Fig. 10. In both the presence and absence of Cl^−^, the reaction proceeds smoothly from reaction (1) to intermediate reaction (4); however, the energy barrier of intermediate reaction (5), in which the OH adsorbed onto the electrocatalyst is desorbed, is large. Additionally, each intermediate is destabilized more in the presence of Cl^−^ than in its absence. This destabilization causes the energy barrier of 10.2 eV for the intermediate reaction (5) in the absence of Cl^−^ to decrease to 6.1 eV, which means that the presence of Cl^−^ promotes the ORR.

## Conclusions

The FePc/rGO nanocomposite without agglomeration has been developed by electrochemically reducing a mixture of completely monolayer GO dispersion and well-dispersed FePc in CHCl_3_. The FePc/rGO nanocomposite exhibited high ORR activity, with an ORR onset and half-wave potential of 0.97 and 0.86 V, respectively, which are greater than those of a commercially available 20 wt% Pt/C catalyst. The high ORR activity is attributed to that the FePc/GO nanocomposite has not only nanolevel structure but also higher electrical conductivity and greater Fe^2+^/Fe^3+^ ratio. The ORR activity could be further improved by adding an electrolyte such as KCl or KNO_3_. The theoretical calculations indicated that the presence of Cl^−^ and NO_3_^−^ ions lowered the conversion energy barrier, supporting the experimental results.

## Conflicts of interest

There are no conflicts to declare.

## Supplementary Material

RA-011-D1RA01001H-s001
