# Supplementary material for: Oxygen reduction reaction activity of an iron phthalocyanine/graphene oxide nanocomposite
Source: RSC Adv. 2021 Apr 29;11(26):15927–32. doi: 10.1039/d1ra01001h (PMC9031042; doi:10.1039/d1ra01001h)
Supplement: RA-011-D1RA01001H-s001 [file RA-011-D1RA01001H-s001.pdf]

Electronic Supplementary Material (ESI) for RSC Advances.
This journal is © The Royal Society of Chemistry 2021

Supplementary information

Oxygen reduction reaction activity of an iron phthalocyanine/graphene oxide nanocomposite

Kenichiro Irisa,[a] Kazuto Hatakeyama,*[b] Soichiro Yoshimoto,[b] Michio Koinuma,*[b] and Shintaro Ida*[b]

a. Graduate School of Science and Technology, Kumamoto University, 2-39-1 Kurokami, Chuo-ku, Kumamoto 860-8555, Japan.
b. Institute of Industrial Nanomaterials (IINa), Kumamoto University, 2-39-1 Kurokami, Chuo-ku, Kumamoto 860-8555, Japan.

Corresponding author
ida-s@kumamoto-u.ac.jp

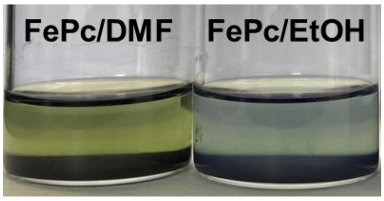

Fig. S1 Photographs of an FePc/ MtOH, FePc/DMF and FePc/$CHCl_3$ dispersion after 1 day.

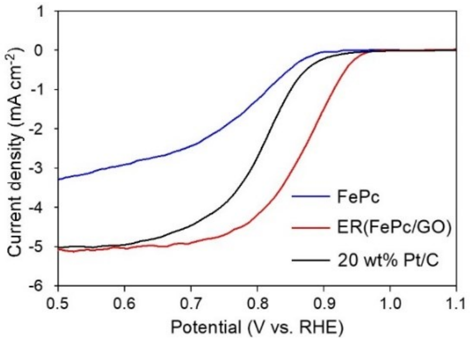

Fig. S2 ORR polarization curves of FePc, ER(FePc/GO), and 20 wt% PtC in O_2-saturated 0.1 M KOH.

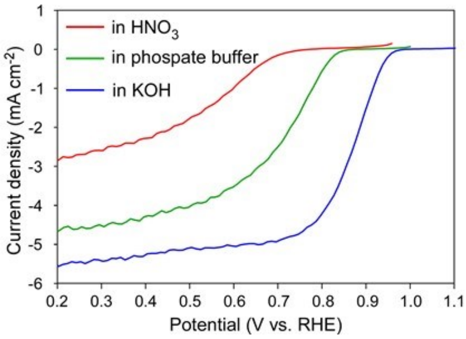

Fig. S3 ORR polarization curves of ER(FePc/GO) in O_2-saturated 0.1 M KOH, phosphate buffer and 0.1 M HNO_3.

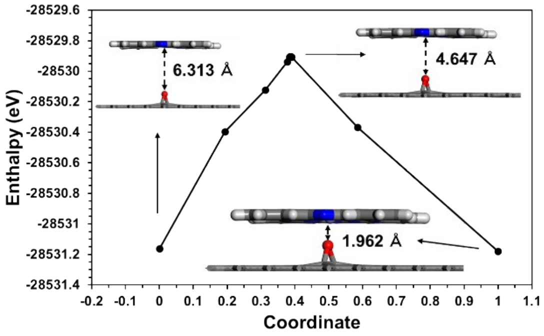

Fig. S4 Optimization of the distance between the Fe atom in FePc and an O atom on a graphene framework.
